# Ascertaining the association between smoking behaviors and viral hepatitis risk: A Mendelian randomization approach

**DOI:** 10.18332/tid/204511

**Published:** 2025-07-26

**Authors:** Birong Lin, Huaxi Ma, Yan Lin, Ting Lin, Xiao Han, Minghua Lin, Haibing Gao

**Affiliations:** 1 Department of Infectious Diseases, Mengchao Hepatobiliary Hospital of Fujian Medical University, Fuzhou, China; 2 College of Biological Science and Engineering, Fuzhou University, Fuzhou, China

**Keywords:** viral hepatitis, smoking behavior, Mendelian randomization, association analysis, public health intervention strategies

## Abstract

**INTRODUCTION:**

Viral hepatitis, caused by various hepatitis viruses, is a global health threat leading to chronic liver disease, hepatic cirrhosis, hepatic failure, and hepatocellular carcinoma. Smoking, a known risk factor for numerous chronic diseases, has been implicated in the pathogenesis of viral hepatitis. However, understanding the relationship between smoking and viral hepatitis is complex due to the presence of confounding factors and the potential for reverse associations.

**METHODS:**

We utilized Mendelian randomization (MR) analysis to explore the potential association between smoking behavior and viral hepatitis. In this study, SNPs were utilized as instrumental variables in a Mendelian randomization framework to examine the relationship between smoking behavior and viral hepatitis risk. To ensure the accuracy of the experiment, our data were sourced from large-scale genome-wide association studies (GWAS) and analyzed using a series of methods, such as inverse variance weighting (IVW) and leave-one-out analysis.

**RESULTS:**

The MR analysis revealed significant positive associations between SNPs related to smoking initiation, status, and cessation, and the risk of viral hepatitis. The IVW method demonstrated a consistent rightward shift of the effect estimates, indicating a potential increase in viral hepatitis risk associated with smoking exposure. Smoking initiation, status, and cessation were associated with increased odds of viral hepatitis by 2.17-fold (95% CI: 1.45–3.24, p=0.00015), 2.93-fold (95% CI: 1.58–5.41, p=0.00061), and 5.30-fold (95% CI: 2.05–13.70, p=0.00057), respectively. The leave-one-out analysis further validated the robustness of our model, with minor SNP-specific deviations observed.

**CONCLUSIONS:**

Our study presents strong associations between smoking behavior and an elevated risk of viral hepatitis, highlighting the need for further investigation into this potential connection. These findings underscore the importance of smoking cessation in liver disease management and inform public health strategies aimed at reducing the burden of viral hepatitis.

## INTRODUCTION

Viral hepatitis, caused by hepatitis A–E viruses, remains a major global public health concern^1^. Each of these viruses differs in its transmission route, clinical course, and potential complications^2-4^. Hepatitis A and E are primarily spread by means of the fecal-oral pathway, while blood and body fluid or perinatal transmission are common modes of transmission for hepatitis B, C, and D^5^. Among them, HBV and HCV are the most prevalent and clinically significant, contributing to a significant portion of chronic liver disease cases globally^6,7^.

According to the World Health Organization (WHO), approximately 250 million people worldwide suffer from hepatitis B^8^, and over 70 million people suffer from hepatitis C^9^. These chronic infections are responsible for severe hepatic disease, including cirrhosis, hepatic failure, and hepatocellular carcinoma (HCC)^10,11^, one of the most common types of liver cancer^12^. Although widespread vaccination and antiviral therapies have substantially improved disease control, particularly for HBV and HCV, the global burden remains high^13^, especially in low-resource regions where diagnosis and treatment are often delayed^14^.

In addition to viral factors, lifestyle^15-17^ elements such as smoking and alcohol consumption play a significant part in liver disease progression. Smoking has been linked not only to cardiovascular^18,19^ and respiratory diseases^20^ but also to liver injury and possibly the progression of viral hepatitis. Emerging research suggests that smoking may also have an impact on the pathogenesis and progression of viral hepatitis, further complicating the disease burden for those infected with hepatitis viruses^21^.

Smoking can exacerbate liver damage and influence the progression of viral hepatitis through multiple mechanisms. First, smoking-related structural changes in the respiratory system, such as airway inflammation, fibrosis, and decreased mucociliary clearance, make it easier for pathogens, including hepatitis viruses, to invade and colonize the body. These respiratory changes, combined with the liver’s role in metabolizing harmful chemicals found in cigarette smoke, generate toxic metabolites that may trigger oxidative stress and inflammatory responses in hepatocytes. Over time, these processes can contribute to liver cell injury, fibrosis, and, eventually, cirrhosis^22^.

Furthermore, smoking may directly influence the replication of hepatitis viruses and impair the host’s immune response^23^. Some studies suggest that smoking can modulate immune cell function, leading to a weakened immune response to viral infections, including those caused by hepatitis viruses^24^. This weakened response may allow the virus to replicate more freely, contributing to a higher viral load and more severe liver damage.

In this study, we employed a two-sample Mendelian randomization approach to explore the potential association between genetically predicted smoking behaviors and the risk of viral hepatitis. Single nucleotide polymorphisms (SNPs) strongly associated with smoking traits –such as nicotine dependence and smoking initiation – were selected as instrumental variables. Through this genetic approach, we aimed to assess whether smoking behavior may have a potential impact on the development or progression of viral hepatitis.

## METHODS

### Study design

**Figure 1:**
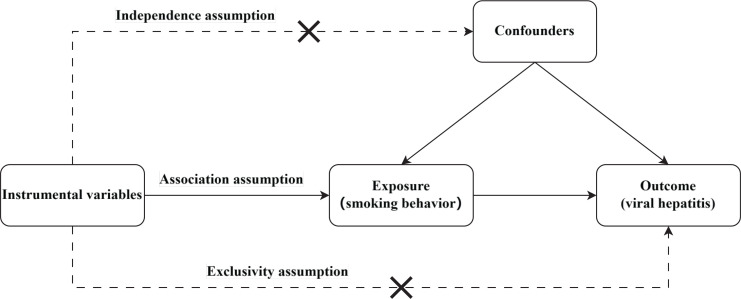
Diagram of MR study design: 1) Association assumption: the instrumental variable is strongly associated with the exposure; 2) Independence assumption: the instrumental variable is independent of confounders; 3) Exclusivity assumption: the instrumental variable affects the outcome only through the exposure

To evaluate the potential association between smoking and viral hepatitis, this study employed Mendelian randomization (MR) analysis. MR analysis utilizes genetic variants as instrumental variables (IVs), typically single nucleotide polymorphisms (SNPs)^25^. The selection of IVs must meet three criteria for valid instrumental variable analysis: 1) The genetic variant is highly correlated with the exposure; only a significant correlation between the IV and the exposure justifies its use as a proxy in MR analysis; 2) the genetic variations have no direct impact on the outcome, otherwise it becomes challenging to discern whether the outcome is caused by the exposure or directly by the IV; and 3) The genetic variant is not associated with confounders^26^ (if the IV is correlated with other factors, the results may reflect the influence of the IV on the outcome through these factors rather than through the exposure, thereby weakening the interpretability of the findings and increasing the risk of misleading conclusions). Therefore, the selection of instrumental variables must strictly adhere to these conditions to ensure the credibility and accuracy of the MR analysis results. The design is shown in Figure 1.

### Data sources

This study is based on large-scale Genome-Wide Association Study (GWAS) data, selecting SNPs significantly associated with smoking behavior as instrumental variables. The GWAS summary genetic data for viral hepatitis were obtained from the Finnish database R10 (https://www.finngen.fi/en), with a total sample size of 409861 individuals. The study used ICD-10 code B15-B19 to define viral hepatitis. GWAS data related to smoking behaviors – including smoking initiation, smoking status, and smoking cessation – were retrieved from the EMBL-EBI database (https://www.ebi.ac.uk/), with the corresponding accession codes being GC-ST007474 (smoking initiation, 1061 SNPs), GC-ST007327 (smoking status, 230 SNPs), and GC-ST007464 (smoking cessation, 82 SNPs). Since all datasets were obtained from publicly available sources, no additional ethical approval was required. Characteristics of exposure and outcome GWAS samples are detailed in Table 1.

**Table 1:** Basic information about the GWAS data used in this study

*Dataset*	*Exposure/Outcome*	*Sample size*	*Year*	*Population*
GCST007474	Smoking initiation	1232091	2019	European
GCST007327	Smoking status	518633	2019	European
GCST007464	Smoking cessation	820192	2019	European
finngen_R10_AB1_VIRAL_HEPATITIS	Viral hepatitis	409861	2023	European

GWAS: genome-wide association study.

### Genetic instrument selection

Throughout the Mendelian randomization analysis, the same criteria were used to determine the genetic instruments for smoking. A genome-wide significance threshold of p<5×10^-8^ was set, and linkage disequilibrium (LD) was excluded (r^2^<0.001; kb>10000). Moreover, we computed the F-statistic for the instrumental variables in order to reduce the bias that may arise from weak instruments. The formula used was F=R^2^(N-2)/(1-R^2^), and we ensured that F>10. Data presentation and analysis were conducted using various charts such as forest plots, scatter plots, funnel plots, and leave-one-out plots. Additionally, the results were robustly tested using multiple methods including univariate MR, multivariate MR, weighted median method, and MR-Egger regression.

### Statistical analysis

The statistical analysis was performed to verify the validity of the chosen IVs and to assess their potential as proxies for smoking behavior. The strength of the association between the IVs and smoking behavior was quantified using odds ratios (ORs) and corresponding 95% confidence intervals (CIs). The I^2^ statistic was used to assess heterogeneity across studies, while Egger’s regression test was applied to evaluate publication bias.

To further validate the robustness of our findings, a series of sensitivity analyses were performed. These included leave-one-out analyses to assess the influence of each individual SNP on the overall MR estimate, and a weighted median analysis to provide an alternative estimate that is less sensitive to outliers. The primary analysis used the inverse-variance weighted (IVW) method under a random-effects model to account for potential heterogeneity.

To evaluate the robustness of the IVW estimate and explore potential horizontal pleiotropy, we performed a series of sensitivity analyses. Funnel plot asymmetry was assessed using Egger’s regression test and the Harbord test. To address possible pleiotropic effects, we applied pleiotropy-robust MR methods, including MR-Egger regression, which accounts for pleiotropy when examining the association between the exposure and outcome. The MR-Egger intercept was used to test for directional pleiotropy, with p<0.05 indicating its presence. We assessed the validity of the InSIDE assumption; a non-significant result supports its validity, justifying the use of MR-Egger in our analysis. All statistical tests were two-sided, and p<0.05 was considered statistically significant. All statistical analyses were conducted in R software (version 4.0.2) with the *MendelianRandomization* package. Custom scripts used for data processing and analysis are available upon request.

## RESULTS

### SNPs linked to smoking show significant associations with viral hepatitis risk

**Figure 2:**
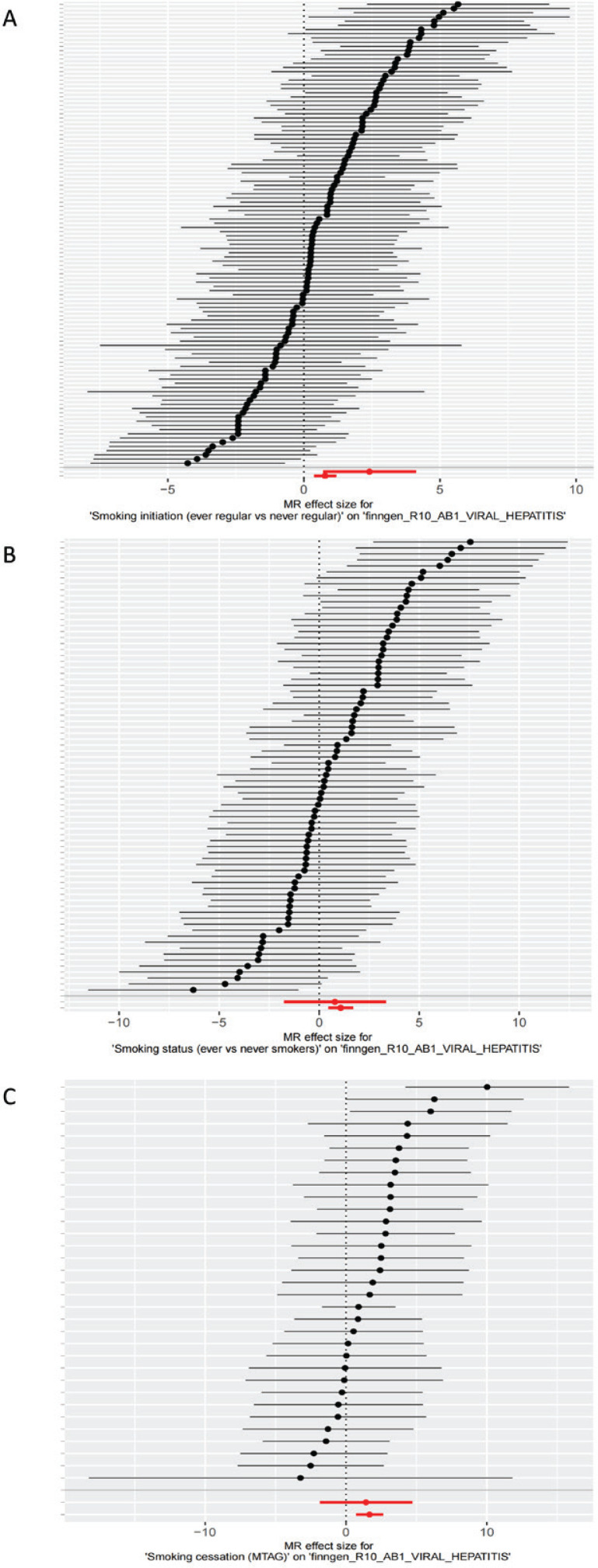
Forest plot of genetic associations between smoking phenotypes and viral hepatitis: A) Smoking initiation for viral hepatitis; B) Smoking status for viral hepatitis; C) Smoking cessation for viral hepatitis

The forest plot (Figure 2) revealed that the majority of SNPs associated with smoking initiation showed a positive association, suggesting a potential positive link between these SNPs and viral hepatitis. A substantial portion of SNPs related to smoking status and cessation also displayed positive associations, with a minority showing negative associations. However, it is important to note that the effect estimates of individual SNPs may contain some error; therefore, we employed the IVW method to synthesize the effect estimates of all SNPs. The red line of the IVW method, which lies entirely to the right of the 0 line, indicates that the composite results support the potential for smoking exposure to elevate the risk of viral hepatitis. The IVW results showed significant associations between smoking initiation (OR=2.17; 95% CI: 0.37–1.18, p=0.00015), smoking status (OR=2.93; 95%CI: 0.46–1.69, p=0.00061), smoking cessation (OR=5.30; 95%CI: 0.72–2.62, p=0.00057), and the risk of viral hepatitis (Figure 2). We concluded that the positive association between smoking initiation, smoking status, and viral hepatitis was supported by multiple SNP instrumental variables.

### Consistent SNP impact on viral hepatitis risk, supporting association analysis

**Figure 3:**
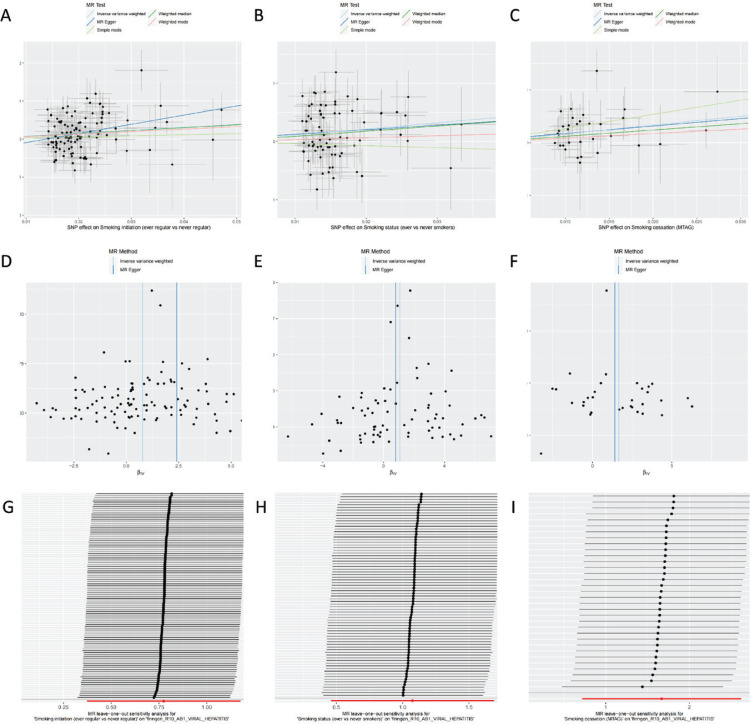
Sensitivity analyses and pleiotropy diagnostics for Mendelian randomization estimates of smoking behaviors on viral hepatitis risk. A–C) Scatter plot of genetic associations between smoking phenotypes and viral hepatitis: A) Smoking initiation for viral hepatitis; B) Smoking status for viral hepatitis; C) Smoking cessation for viral hepatitis. D–F) Funnel plot of genetic associations between smoking phenotypes and viral hepatitis: D) Smoking initiation for viral hepatitis; E) Smoking status for viral hepatitis; F) Smoking cessation for viral hepatitis. G–I) Leave-one-out of genetic associations between smoking phenotypes and viral hepatitis: G) Smoking initiation for viral hepatitis; H) Smoking status for viral hepatitis; I) Smoking cessation for viral hepatitis

In this study, scatter plots (Figure 3) were used to visualize the relationship between SNPs and the effect estimates of smoking initiation, smoking status, smoking cessation, and viral hepatitis. Most SNPs clustered closely around the trend line, suggesting consistent effect directions and magnitudes across exposures. The IVW-derived trend lines showed a positive slope in all three plots, indicating a potential positive association between smoking-related exposures and viral hepatitis. No substantial outliers or evidence of pleiotropy were observed, reinforcing the robustness of SNPs as instrumental variables. A few SNPs deviated notably from the trend line, which may reflect specific effects or unaccounted confounding (Figure 3). These findings are consistent with those from the forest plots and support the reliability of the analysis.

### Minimal publication bias in MR analysis

In the Mendelian randomization analysis of this study, we employed a funnel plot to assess the possibility of publication bias and small sample effects. The funnel plot (Figure 3) displays the relationship between the SNP effect estimates and their standard errors, and the observed scatter of effect estimates against standard errors was roughly symmetrical, indicating that the distribution of SNPs in the dataset was relatively uniform and the precision of effect estimates was consistent. We noticed two significant deviations on the right side of the funnel plot, which may reflect outliers in the effect estimates of a few SNPs or other potential influencing factors. However, no significant bias was observed overall, suggesting that our analysis results are relatively reliable and not significantly affected by publication bias (Figure 3). Therefore, the funnel plot analysis supports our research conclusions, confirming the credibility and effectiveness of the methods and data used in the Mendelian randomization analysis.

### Model robustness, with minor SNP-specific deviations

In this study, we conducted Mendelian randomization analysis and used a leave-one-out plot (Figure 3) to display the effect estimate results after excluding individual SNPs. The leave-one-out analysis suggested that the IVW analysis results of the remaining SNPs were consistent with the analysis results that included all SNPs, with no significant differences in statistical results (p>0.05). It can be observed that the effect estimates of almost all excluded SNPs were concentrated on the right side of the 0 line, consistently indicating a positive association between smoking initiation, smoking status, and smoking cessation as exposure variables and viral hepatitis as the outcome (Figure 3). The overall effect estimate remained relatively stable after the removal of different SNPs, indicating that our model was not sensitive to the exclusion of individual SNPs. This consistent result provides strong support for the robustness of our model and significantly enhances our confidence in the effectiveness of SNPs as instrumental variables in identifying the association between smoking exposure and the onset of viral hepatitis. However, we also noticed that a few individual points showed deviations, especially the smoking cessation-related SNP, rs12378015, indicating that this SNP may have a considerable effect on the development of chronic hepatitis. This deviation is consistent with its larger confidence interval. Therefore, the analysis of the leave-one-out plot reveals the potential positive impact of smoking on the risk of viral hepatitis and validates the robustness of our model and the rational use of SNPs as effective instrumental variables.

## DISCUSSION

This study used Mendelian randomization to explore whether genetically predicted smoking behaviors, including initiation, status, and cessation, are associated with the risk of viral hepatitis. The results indicate a significant positive association, suggesting that smoking behavior is likely a contributing factor to the development of viral hepatitis. Smoking initiation and continuing smoking were both found to significantly increase the risk of viral hepatitis, while the impact of smoking cessation on the risk presented complexity and variability. These findings were substantiated by various statistical methods, including the IVW method and leave-one-out analysis, supporting an association between smoking and the onset of viral hepatitis.

The harmful chemicals in tobacco, such as nicotine and polycyclic aromatic hydrocarbons, generate toxic metabolites after liver metabolism, leading to oxidative stress and inflammatory responses in hepatocytes, which can cause liver damage^27,28^. Smoking may also suppress immune function, reducing the body’s resistance to hepatitis viruses and increasing the risk of infection^29^. Furthermore, chronic inflammation due to long-term smoking may promote liver fibrosis and other pathological changes^30,31^, accelerating the progression of viral hepatitis. During the smoking cessation process, the body undergoes adaptation and regulation, which could lead to temporary immune system disruptions and changes in liver metabolic functions, thus having complex effects on viral infections^32^.

In the Mendelian randomization analysis, we utilized SNPs highly associated with smoking behavior as instrumental variables to explore their impact on the risk of viral hepatitis. The effect estimates of the majority of SNPs showed a positive association with viral hepatitis, indicating that these SNPs might indirectly affect liver health by regulating smoking behavior. For instance, certain SNPs related to smoking initiation may modulate the rate of nicotine metabolism, affecting an individual’s level of tobacco exposure and consequently increasing the toxic burden on the liver. Additionally, among SNPs related to smoking cessation, one SNP (rs12378015) demonstrated a significant association with the risk of viral hepatitis, suggesting its potential role in modulating immune responses and metabolic pathways during the smoking cessation process, thereby affecting liver health. These genetic variant studies provide new clues for further understanding the genetic basis between smoking behavior and liver disease risk.

### Strengths and limitations

We employed Mendelian randomization (MR) to investigate the association between smoking behavior and viral hepatitis. This approach utilizes genetic variants as instrumental variables, effectively mitigating confounding biases and reverse causation inherent in conventional observational studies. The smoking-associated genetic instruments were derived from large-scale genome-wide association studies (GWAS), with genome-wide significant associations (p<5×10^-8^) ensuring instrument validity. However, it should be noted that our research has certain limitations. First, the GWAS summary statistics used in our analysis were predominantly derived from European populations, which may limit the generalizability of the findings to other ethnic groups. Second, we did not apply additional pleiotropy-robust methods such as MR-PRESSO, which could help identify and correct for potential outlier variants, and thus we cannot completely rule out the presence of residual horizontal pleiotropy. In addition, genetic variants associated with certain potential confounders, such as alcohol consumption or metabolic traits, were not systematically excluded, and future studies may benefit from more refined instrument selection. It is also worth noting that the outcome variable – viral hepatitis – was analyzed in binary form, which limits exploration of potential dose-response relationships or disease severity gradients. Therefore, while our results provide genetic evidence for associations between smoking-related behaviors and viral hepatitis, they should be interpreted with appropriate caution.

### Future research

Future research should further expand the sample size and integrate more environmental variables (such as alcohol consumption, metabolic syndrome, etc.) to more comprehensively assess the relationship between smoking and viral hepatitis. In addition, indepth biological research on the modes of action of related SNPs will help reveal the specific pathways through which smoking behavior affects liver health and provide a stronger basis for personalized intervention measures. Further studies incorporating multi-ethnic data, stratified analyses, and pleiotropyrobust methodologies are warranted to enhance the robustness and applicability of the findings.

## CONCLUSIONS

This study provides evidence of a potential association between smoking and an increased risk of viral hepatitis, supporting the development of public health intervention strategies and informing future research directions.

## Supplementary Material



## Data Availability

The GWAS summary statistics for smoking behaviors were obtained from the GWAS Catalog (https://www.ebi.ac.uk/gwas/). Viral hepatitis data were from the FinnGen database download (https://www.finngen.fi/en). All datasets are publicly available and require no special access permissions.
