# Supplementary material for: Ascertaining the association between smoking behaviors and viral hepatitis risk: A Mendelian randomization approach
Source: Tob Induc Dis. 2025 Jul 26;23:10.18332/tid/204511. doi: 10.18332/tid/204511 (PMC12305554; doi:10.18332/tid/204511)
Supplement: Supplementary file 1 [file TID-23-105-s1.pdf]

Instrument Variables for MR analysis of smoking initiation and viral hepatitis

SNP	exposure	beta.exposure	se.exposure	pval.exposure	outcome	beta.outcome	se.outcome	pval.outcome
rs1022376	Smoking initiation	-0.014748839	0.00275510204081633	2e-09	finngen_R10_AB1_VIRAL_HEPATITIS	-0.0623526	0.0298118	0.0364796
rs10279261	Smoking initiation	-0.018872635	0.00255102040816327	6e-13	finngen_R10_AB1_VIRAL_HEPATITIS	0.0374587	0.0310635	0.227866
rs10466419	Smoking initiation	-0.019562634	0.00331632653061225	4e-09	finngen_R10_AB1_VIRAL_HEPATITIS	-0.0263505	0.0362787	0.467634
rs10508047	Smoking initiation	-0.014830017	0.00255102040816327	1e-08	finngen_R10_AB1_VIRAL_HEPATITIS	-0.0142552	0.029796	0.632346
rs10698713	Smoking initiation	-0.03351702	0.00561224489795918	2e-09	finngen_R10_AB1_VIRAL_HEPATITIS	0.02871	0.113433	0.80019
rs10789369	Smoking initiation	-0.023448382	0.00280612244897959	3e-19	finngen_R10_AB1_VIRAL_HEPATITIS	-0.04111	0.030913	0.183564
rs10885460	Smoking initiation	-0.018677495	0.00280612244897959	4e-11	finngen_R10_AB1_VIRAL_HEPATITIS	0.0448939	0.032378	0.165577
rs10914684	Smoking initiation	-0.015803844	0.00280612244897959	6e-09	finngen_R10_AB1_VIRAL_HEPATITIS	0.0224205	0.0315289	0.477016
rs10960092	Smoking initiation	-0.020486193	0.00280612244897959	1e-12	finngen_R10_AB1_VIRAL_HEPATITIS	-0.00522023	0.0315179	0.86845
rs11057005	Smoking initiation	-0.015713902	0.00255102040816327	8e-10	finngen_R10_AB1_VIRAL_HEPATITIS	0.0551682	0.0299662	0.0656191
rs1109480	Smoking initiation	-0.016691703	0.00255102040816327	2e-10	finngen_R10_AB1_VIRAL_HEPATITIS	-0.0472945	0.031336	0.13123
rs11320003	Smoking initiation	-0.018876148	0.00306122448979592	1e-10	finngen_R10_AB1_VIRAL_HEPATITIS	0.0633507	0.0365628	0.0831572
rs1138997	Smoking initiation	-0.020487192	0.00280612244897959	2e-13	finngen_R10_AB1_VIRAL_HEPATITIS	-0.0934109	0.0311654	0.00272414
rs1150668	Smoking initiation	-0.018510304	0.00280612244897959	3e-11	finngen_R10_AB1_VIRAL_HEPATITIS	-0.00570354	0.0299001	0.848719
rs11587399	Smoking initiation	-0.017804557	0.00306122448979592	7e-09	finngen_R10_AB1_VIRAL_HEPATITIS	-0.0585076	0.0397345	0.140896
rs11642231	Smoking initiation	-0.015988343	0.00280612244897959	3e-09	finngen_R10_AB1_VIRAL_HEPATITIS	-0.00740169	0.0299785	0.804983
rs117657830	Smoking initiation	-0.037759688	0.00637755102040816	3e-09	finngen_R10_AB1_VIRAL_HEPATITIS	0.0668675	0.119108	0.574522
rs11681481	Smoking initiation	-0.018556055	0.00280612244897959	5e-12	finngen_R10_AB1_VIRAL_HEPATITIS	0.0387575	0.0300448	0.197053
rs11783093	Smoking initiation	-0.047124278	0.00267857142857143	3e-69	finngen_R10_AB1_VIRAL_HEPATITIS	-0.0765518	0.0451202	0.0897677
rs118202	Smoking initiation	-0.03674841	0.00331632653061225	2e-28	finngen_R10_AB1_VIRAL_HEPATITIS	-0.0447372	0.0328267	0.172935
rs11872397	Smoking initiation	-0.017113948	0.00306122448979592	2e-08	finngen_R10_AB1_VIRAL_HEPATITIS	0.0447853	0.0361834	0.215816
rs118720	Smoking initiation	-0.014721156	0.00255102040816327	5e-08	finngen_R10_AB1_VIRAL_HEPATITIS	-0.00550853	0.0307522	0.509555
rs11889814	Smoking initiation	-0.021026509	0.0038265306122449	3e-08	finngen_R10_AB1_VIRAL_HEPATITIS	0.0508862	0.0434337	0.241365
rs12027999	Smoking initiation	-0.024358766	0.0038265306122449	1e-10	finngen_R10_AB1_VIRAL_HEPATITIS	-0.0558236	0.0371223	0.13264
rs12130857	Smoking initiation	-0.018003138	0.00255102040816327	4e-11	finngen_R10_AB1_VIRAL_HEPATITIS	0.00471327	0.0330156	0.886481
rs12530388	Smoking initiation	-0.018306018	0.00255102040816327	2e-12	finngen_R10_AB1_VIRAL_HEPATITIS	-0.00452468	0.0299682	0.879941
rs12633090	Smoking initiation	-0.023019686	0.00255102040816327	2e-12	finngen_R10_AB1_VIRAL_HEPATITIS	-0.0546036	0.0443996	0.218764
rs12642744	Smoking initiation	-0.016590752	0.00280612244897959	4e-09	finngen_R10_AB1_VIRAL_HEPATITIS	0.00710613	0.0390418	0.855572
rs12739922	Smoking initiation	-0.021258139	0.00255102040816327	4e-12	finngen_R10_AB1_VIRAL_HEPATITIS	-0.0326456	0.0327902	0.328425
rs12755632	Smoking initiation	-0.015405137	0.00280612244897959	4e-08	finngen_R10_AB1_VIRAL_HEPATITIS	-0.0331706	0.0313649	0.290251
rs12918191	Smoking initiation	-0.017927034	0.00306122448979592	4e-09	finngen_R10_AB1_VIRAL_HEPATITIS	0.0011584	0.0390119	0.976319
rs13100073	Smoking initiation	-0.024642577	0.00255102040816327	5e-22	finngen_R10_AB1_VIRAL_HEPATITIS	-0.0223073	0.0300543	0.457946
rs13237637	Smoking initiation	-0.023682335	0.00255102040816327	2e-20	finngen_R10_AB1_VIRAL_HEPATITIS	0.0403443	0.0297826	0.175536
rs13261666	Smoking initiation	-0.019994581	0.00255102040816327	4e-10	finngen_R10_AB1_VIRAL_HEPATITIS	0.0427823	0.0298221	0.156655
rs13392227	Smoking initiation	-0.023437547	0.0038265306122449	2e-10	finngen_R10_AB1_VIRAL_HEPATITIS	0.0571204	0.0377731	0.322745
rs13437771	Smoking initiation	-0.027109828	0.00357142857142857	1e-14	finngen_R10_AB1_VIRAL_HEPATITIS	0.00090873	0.0356253	0.97965
rs1373178	Smoking initiation	-0.020316398	0.00255102040816327	4e-15	finngen_R10_AB1_VIRAL_HEPATITIS	-0.0436109	0.0308303	0.157201
rs1381775	Smoking initiation	-0.015614667	0.00280612244897959	3e-08	finngen_R10_AB1_VIRAL_HEPATITIS	0.0335029	0.0331961	0.312844
rs13906	Smoking initiation	-0.024529729	0.00408163265306122	2e-09	finngen_R10_AB1_VIRAL_HEPATITIS	-0.0716277	0.0435608	0.100111
rs1449012	Smoking initiation	-0.015373412	0.00255102040816327	2e-09	finngen_R10_AB1_VIRAL_HEPATITIS	0.0679636	0.0307736	0.0272095
rs1514116	Smoking initiation	-0.019300157	0.00280612244897959	8e-14	finngen_R10_AB1_VIRAL_HEPATITIS	-0.0731744	0.0298153	0.0141156
rs1549979	Smoking initiation	-0.024522314	0.00280612244897959	9e-21	finngen_R10_AB1_VIRAL_HEPATITIS	-0.0651915	0.0326588	0.0459187
rs160631	Smoking initiation	-0.017262844	0.00280612244897959	8e-10	finngen_R10_AB1_VIRAL_HEPATITIS	0.0317937	0.0328113	0.332552
rs16826827	Smoking initiation	-0.022268888	0.0038265306122449	1e-08	finngen_R10_AB1_VIRAL_HEPATITIS	-0.0191752	0.0474883	0.686368
rs1714521	Smoking initiation	-0.01629531	0.00255102040816327	2e-10	finngen_R10_AB1_VIRAL_HEPATITIS	-0.0452124	0.0301058	0.133152
rs17197663	Smoking initiation	-0.021587353	0.0038265306122449	2e-08	finngen_R10_AB1_VIRAL_HEPATITIS	-0.10712	0.0527998	0.0424786
rs17229285	Smoking initiation	-0.015479556	0.00280612244897959	4e-08	finngen_R10_AB1_VIRAL_HEPATITIS	-0.0540458	0.0298894	0.0707228
rs1772572	Smoking initiation	-0.016868144	0.00255102040816327	4e-11	finngen_R10_AB1_VIRAL_HEPATITIS	-0.0165374	0.0312463	0.596624
rs1935571	Smoking initiation	-0.015720382	0.00255102040816327	7e-10	finngen_R10_AB1_VIRAL_HEPATITIS	0.0161782	0.0297557	0.586647
rs2063976	Smoking initiation	-0.020181425	0.00255102040816327	3e-15	finngen_R10_AB1_VIRAL_HEPATITIS	-0.0224289	0.0302593	0.458558
rs2196356	Smoking initiation	-0.018772762	0.00280612244897959	2e-11	finngen_R10_AB1_VIRAL_HEPATITIS	-0.0358197	0.0357519	0.316392
rs2279829	Smoking initiation	-0.017376566	0.00255102040816327	1e-11	finngen_R10_AB1_VIRAL_HEPATITIS	-0.0374078	0.0328015	0.254107
rs2289791	Smoking initiation	-0.017725516	0.00306122448979592	6e-09	finngen_R10_AB1_VIRAL_HEPATITIS	0.00459248	0.0367271	0.900489
rs2587507	Smoking initiation	-0.01466024	0.00255102040816327	9e-09	finngen_R10_AB1_VIRAL_HEPATITIS	-0.00623261	0.0297887	0.834271
rs2710634	Smoking initiation	-0.017761428	0.00255102040816327	4e-12	finngen_R10_AB1_VIRAL_HEPATITIS	-0.0567388	0.0299144	0.0578669
rs28441558	Smoking initiation	-0.03556498	0.00357142857142857	1e-23	finngen_R10_AB1_VIRAL_HEPATITIS	-0.0874499	0.0623549	0.160779
rs2901785	Smoking initiation	-0.017308272	0.00255102040816327	1e-11	finngen_R10_AB1_VIRAL_HEPATITIS	0.011465	0.0298947	0.701339
rs2939756	Smoking initiation	-0.015699685	0.00255102040816327	7e-10	finngen_R10_AB1_VIRAL_HEPATITIS	0.00665739	0.0297842	0.82313
rs3115418	Smoking initiation	-0.01422488	0.00255102040816327	3e-08	finngen_R10_AB1_VIRAL_HEPATITIS	0.0142639	0.0297608	0.631735
rs3172494	Smoking initiation	-0.029126683	0.00331632653061224	3e-18	finngen_R10_AB1_VIRAL_HEPATITIS	0.0308873	0.0361016	0.392236
rs3218116	Smoking initiation	-0.019843036	0.00306122448979592	1e-11	finngen_R10_AB1_VIRAL_HEPATITIS	-0.0069877	0.0347428	0.8406
rs329124	Smoking initiation	-0.016387068	0.00255102040816327	1e-10	finngen_R10_AB1_VIRAL_HEPATITIS	0.0264098	0.0301197	0.38058
rs34342129	Smoking initiation	-0.014281006	0.00255102040816327	2e-08	finngen_R10_AB1_VIRAL_HEPATITIS	0.00167683	0.0297496	0.955051
rs35375873	Smoking initiation	-0.02701025	0.00408163265306123	3e-11	finngen_R10_AB1_VIRAL_HEPATITIS	-0.0278096	0.0397978	0.484694
rs3764351	Smoking initiation	-0.014749162	0.00267857142857143	4e-08	finngen_R10_AB1_VIRAL_HEPATITIS	-0.0635391	0.0319616	0.0468134
rs3820277	Smoking initiation	-0.016836917	0.00255102040816327	2e-13	finngen_R10_AB1_VIRAL_HEPATITIS	0.00239064	0.0298419	0.936149
rs3934797	Smoking initiation	-0.021297466	0.00331632653061225	1e-10	finngen_R10_AB1_VIRAL_HEPATITIS	-0.0549869	0.0413644	0.183739
rs4044321	Smoking initiation	-0.022640966	0.00280612244897959	2e-17	finngen_R10_AB1_VIRAL_HEPATITIS	-0.0678107	0.0313101	0.0303284
rs4148932	Smoking initiation	-0.014044522	0.0025765306122449	4e-08	finngen_R10_AB1_VIRAL_HEPATITIS	-0.0146814	0.0297548	0.645637
rs4310804	Smoking initiation	-0.018187258	0.00306122448979592	8e-10	finngen_R10_AB1_VIRAL_HEPATITIS	0.0257997	0.0324303	0.426297
rs4326350	Smoking initiation	-0.017613662	0.00255102040816327	5e-12	finngen_R10_AB1_VIRAL_HEPATITIS	-0.0371795	0.0307566	0.226728
rs4462653	Smoking initiation	-0.018486165	0.00280612244897959	5e-11	finngen_R10_AB1_VIRAL_HEPATITIS	-0.0179298	0.0309817	0.618271
rs4674993	Smoking initiation	-0.024005337	0.00306122448979592	4e-15	finngen_R10_AB1_VIRAL_HEPATITIS	-0.0517273	0.0393932	0.0193135
rs4818005	Smoking initiation	-0.020430982	0.00280612244897959	4e-14	finngen_R10_AB1_VIRAL_HEPATITIS	0.0209817	0.0323002	0.517962
rs4822102	Smoking initiation	-0.016542807	0.00280612244897959	3e-09	finngen_R10_AB1_VIRAL_HEPATITIS	-0.0199139	0.0297937	0.503883
rs4837631	Smoking initiation	-0.015357429	0.00255102040816327	2e-09	finngen_R10_AB1_VIRAL_HEPATITIS	0.0602761	0.0298155	0.0432146
rs4877285	Smoking initiation	-0.018131824	0.00255102040816327	2e-11	finngen_R10_AB1_VIRAL_HEPATITIS	-0.00182145	0.0316899	0.954165
rs55944129	Smoking initiation	-0.017565375	0.00306122448979592	1e-08	finngen_R10_AB1_VIRAL_HEPATITIS	-0.00417495	0.0322822	0.897099
rs58400863	Smoking initiation	-0.020171883	0.00255102040816327	3e-15	finngen_R10_AB1_VIRAL_HEPATITIS	-0.00584538	0.031086	0.850936
rs586699	Smoking initiation	-0.014803281	0.00255102040816327	7e-09	finngen_R10_AB1_VIRAL_HEPATITIS	0.00826962	0.0297418	0.782402
rs6011779	Smoking initiation	-0.01917655	0.00331632653061224	3e-09	finngen_R10_AB1_VIRAL_HEPATITIS	-0.0232692	0.0345485	0.500615
rs6073075	Smoking initiation	-0.018704044	0.00255102040816327	2e-13	finngen_R10_AB1_VIRAL_HEPATITIS	-0.060216	0.0421288	0.152909
rs61886926	Smoking initiation	-0.017940067	0.00255102040816327	2e-12	finngen_R10_AB1_VIRAL_HEPATITIS	-0.0859673	0.0300732	0.0042551
rs61959481	Smoking initiation	-0.0203442	0.00306122448979592	2e-11	finngen_R10_AB1_VIRAL_HEPATITIS	0.0490573	0.0387922	0.206009
rs62007780	Smoking initiation	-0.015913295	0.00255102040816327	5e-10	finngen_R10_AB1_VIRAL_HEPATITIS	-0.0534857	0.0304209	0.0797132
rs62052916	Smoking initiation	-0.031913772	0.00510204081632653	4e-10	finngen_R10_AB1_VIRAL_HEPATITIS	-0.186064	0.0540046	0.00089778
rs62106258	Smoking initiation	-0.04549801	0.00586734693877551	9e-15	finngen_R10_AB1_VIRAL_HEPATITIS	0.0186985	0.107503	0.86123
rs62137126	Smoking initiation	-0.023691086	0.0038265306122449	1e-09	finngen_R10_AB1_VIRAL_HEPATITIS	-0.0132955	0.0497713	0.789132
rs62180324	Smoking initiation	-0.019516665	0.00331632653061224	4e-09	finngen_R10_AB1_VIRAL_HEPATITIS	-0.0287759	0.0411996	0.484895
rs6265	Smoking initiation	-0.029275492	0.00331632653061224	3e-19	finngen_R10_AB1_VIRAL_HEPATITIS	-0.0489878	0.0413796	0.236467
rs644740	Smoking initiation	-0.014078517	0.00255102040816327	4e-08	finngen_R10_AB1_VIRAL_HEPATITIS	0.075983	0.03048	0.0190003
rs6474609	Smoking initiation	-0.015588739	0.00255102040816327	6e-10	finngen_R10_AB1_VIRAL_HEPATITIS	0.0135279	0.0297975	0.649832
rs67050670	Smoking initiation	-0.020272274	0.00306122448979592	2e-11	finngen_R10_AB1_VIRAL_HEPATITIS	-0.00188609	0.0368501	0.95918
rs6756212	Smoking initiation	-0.03388805	0.00255102040816327	3e-40	finngen_R10_AB1_VIRAL_HEPATITIS	-0.0206105	0.0299672	0.4916
rs67777803	Smoking initiation	-0.024601495	0.00331632653061225	3e-13	finngen_R10_AB1_VIRAL_HEPATITIS	-0.0847793	0.0400097	0.0340934
rs6890961	Smoking initiation	-0.019310867	0.00255102040816327	2e-13	finngen_R10_AB1_VIRAL_HEPATITIS	0.00754308	0.0310606	0.808121
rs6968380	Smoking initiation	-0.023419237	0.00280612244897959	1e-17	finngen_R10_AB1_VIRAL_HEPATITIS	-0.00389254	0.0307279	0.899195
rs6986430	Smoking initiation	-0.024337659	0.00306122448979592	2e-15	finngen_R10_AB1_VIRAL_HEPATITIS	-0.0438621	0.0374936	0.242059
rs7026534	Smoking initiation	-0.016603015	0.00280612244897959	3e-09	finngen_R10_AB1_VIRAL_HEPATITIS	-0.0434632	0.033768	0.198055
rs7072776	Smoking initiation	-0.021974666	0.00280612244897959	6e-15	finngen_R10_AB1_VIRAL_HEPATITIS	-0.00651795	0.0348882	0.851799
rs7134009	Smoking initiation	-0.015796907	0.00280612244897959	4e-08	finngen_R10_AB1_VIRAL_HEPATITIS	0.0110508	0.0310692	0.722078
rs71602617	Smoking initiation	-0.017765487	0.00306122448979592	2e-08	finngen_R10_AB1_VIRAL_HEPATITIS	-0.0694452	0.0325165	0.0327047
rs72780746	Smoking initiation	-0.025762958	0.00331632653061225	2e-14	finngen_R10_AB1_VIRAL_HEPATITIS	-0.0682991	0.0412495	0.097771
rs73008357	Smoking initiation	-0.022309521	0.00408163265306122	2e-08	finngen_R10_AB1_VIRAL_HEPATITIS	0.0665792	0.0473054	0.159299
rs7333559	Smoking initiation	-0.023212526	0.00306122448979592	6e-14	finngen_R10_AB1_VIRAL_HEPATITIS	-0.118924	0.038956	0.00226741
rs75210106	Smoking initiation	-0.01865936	0.00331632653061225	2e-08	finngen_R10_AB1_VIRAL_HEPATITIS	0.0213477	0.03231	0.508795
rs75674569	Smoking initiation	-0.025337664	0.00433673469387755	3e-09	finngen_R10_AB1_VIRAL_HEPATITIS	-0.0101182	0.0634407	0.873282
rs75919030	Smoking initiation	-0.020966403	0.00306122448979592	3e-13	finngen_R10_AB1_VIRAL_HEPATITIS	0.0506269	0.0339861	0.13632
rs7598402	Smoking initiation	-0.014728254	0.00255102040816327	7e-09	finngen_R10_AB1_VIRAL_HEPATITIS	0.0188603	0.0297437	0.52602
rs7600835	Smoking initiation	-0.015121582	0.00267857142857143	2e-08	finngen_R10_AB1_VIRAL_HEPATITIS	0.00864222	0.0333189	0.795343
rs7640107	Smoking initiation	-0.014186049	0.0025765306122449	3e-08	finngen_R10_AB1_VIRAL_HEPATITIS	-0.00225956	0.0297457	0.939449
rs76608582	Smoking initiation	-0.034549292	0.00586734693877551	5e-09	finngen_R10_AB1_VIRAL_HEPATITIS	-0.0295185	0.064012	0.644699
rs77215829	Smoking initiation	-0.024044668	0.00357142857142857	2e-10	finngen_R10_AB1_VIRAL_HEPATITIS	-0.0203885	0.0371461	0.583093
rs77283305	Smoking initiation	-0.015195972	0.00275510204081633	4e-08	finngen_R10_AB1_VIRAL_HEPATITIS	0.0215077	0.0332416	0.517625
rs7809303	Smoking initiation	-0.021418838	0.00280612244897959	3e-15	finngen_R10_AB1_VIRAL_HEPATITIS	0.0518184	0.0315377	0.10037
rs78239456	Smoking initiation	-0.018495236	0.00280612244897959	9e-12	finngen_R10_AB1_VIRAL_HEPATITIS	0.00477349	0.029897	0.873145
rs7836565	Smoking initiation	-0.015508417	0.00283163265306122	4e-08	finngen_R10_AB1_VIRAL_HEPATITIS	0.0559249	0.0323332	0.0836951
rs7901883	Smoking initiation	-0.019257454	0.00306122448979592	2e-10	finngen_R10_AB1_VIRAL_HEPATITIS	0.0822637	0.0350178	0.018814
rs7920501	Smoking initiation	-0.015516845	0.00255102040816327	1e-09	finngen_R10_AB1_VIRAL_HEPATITIS	-0.0428024	0.0304178	0.159382
rs7921378	Smoking initiation	-0.023313547	0.00255102040816327	6e-20	finngen_R10_AB1_VIRAL_HEPATITIS	0.0254429	0.0297575	0.392546
rs7943721	Smoking initiation	-0.02121361	0.00331632653061225	4e-10	finngen_R10_AB1_VIRAL_HEPATITIS	0.0475287	0.0410674	0.247135
rs7969559	Smoking initiation	-0.017015876	0.00306122448979592	2e-09	finngen_R10_AB1_VIRAL_HEPATITIS	-0.0813204	0.0305664	0.00780351
rs876793	Smoking initiation	-0.0179245	0.00255102040816327	6e-11	finngen_R10_AB1_VIRAL_HEPATITIS	-0.0331272	0.0335946	0.32409
rs9323328	Smoking initiation	-0.01423703	0.00255102040816327	3e-08	finngen_R10_AB1_VIRAL_HEPATITIS	-0.0475428	0.0298408	0.111112
rs9423279	Smoking initiation	-0.018580785	0.00280612244897959	3e-12	finngen_R10_AB1_VIRAL_HEPATITIS	-0.00404497	0.0303966	0.894136
rs9540731	Smoking initiation	-0.017730324	0.00255102040816327	3e-12	finngen_R10_AB1_VIRAL_HEPATITIS	-0.0171418	0.0299029	0.566477
rs9545155	Smoking initiation	-0.01607088	0.00255102040816327	3e-10	finngen_R10_AB1_VIRAL_HEPATITIS	-0.0024644	0.0297741	0.934034
rs9627272	Smoking initiation	-0.015473696	0.00280612244897959	2e-09	finngen_R10_AB1_VIRAL_HEPATITIS	0.00822004	0.031856	0.796378
rs9826984	Smoking initiation	-0.014052859	0.0025765306122449	4e-08	finngen_R10_AB1_VIRAL_HEPATITIS	0.00227574	0.0297422	0.939009
rs9922607	Smoking initiation	-0.022159314	0.00306122448979592	3e-12	finngen_R10_AB1_VIRAL_HEPATITIS	-0.0954498	0.0553398	0.0845649
rs9941217	Smoking initiation	-0.01855667	0.00280612244897959	4e-12	finngen_R10_AB1_VIRAL_HEPATITIS	0.0072452	0.0314717	0.817926

Instrument Variables for MR analysis of Smoking status and viral hepatitis

SNP	exposure	beta.exposure	se.exposure	pval.exposure	outcome	beta.outcome	se.outcome	pval.outcome
rs1022261	Smoking status (ev	-0.011013538	0.00201530612244898	4e-08	finngen_R10_AB1_VIRAL_HEPATITIS	-0.03523	0.0298096	0.237272
rs10252114	Smoking status (ev	-0.01233972	0.00221938775510204	3e-08	finngen_R10_AB1_VIRAL_HEPATITIS	-0.0640359	0.0303138	0.0346497
rs10751226	Smoking status (ev	-0.014731288	0.0026530612244898	3e-08	finngen_R10_AB1_VIRAL_HEPATITIS	0.0526365	0.0407114	0.196039
rs10914684	Smoking status (ev	-0.015183688	0.00204081632653061	1e-12	finngen_R10_AB1_VIRAL_HEPATITIS	0.0224205	0.0315289	0.477016
rs10952199	Smoking status (ev	-0.013566539	0.00219387755102041	6e-10	finngen_R10_AB1_VIRAL_HEPATITIS	0.0271665	0.0299881	0.364984
rs10970595	Smoking status (ev	-0.018772665	0.00306122448979592	2e-09	finngen_R10_AB1_VIRAL_HEPATITIS	-0.0727629	0.0504033	0.148848
rs11066971	Smoking status (ev	-0.016561227	0.00280612244897959	6e-09	finngen_R10_AB1_VIRAL_HEPATITIS	0.0200892	0.043423	0.643622
rs1109480	Smoking status (ev	-0.013624338	0.00221938775510204	9e-10	finngen_R10_AB1_VIRAL_HEPATITIS	-0.0472945	0.031336	0.13123
rs11165528	Smoking status (ev	-0.012844953	0.00216836734693878	3e-09	finngen_R10_AB1_VIRAL_HEPATITIS	0.0157549	0.0297669	0.596615
rs11210892	Smoking status (ev	-0.015572052	0.00229591836734694	4e-13	finngen_R10_AB1_VIRAL_HEPATITIS	-0.0484842	0.0316421	0.125456
rs11255908	Smoking status (ev	-0.01461856	0.00229591836734694	2e-10	finngen_R10_AB1_VIRAL_HEPATITIS	0.00980072	0.0408414	0.810353
rs1136998	Smoking status (ev	-0.020011662	0.00306122448979592	1e-09	finngen_R10_AB1_VIRAL_HEPATITIS	-0.0611142	0.0553817	0.269806
rs11771982	Smoking status (ev	-0.01631134	0.00280612244897959	5e-09	finngen_R10_AB1_VIRAL_HEPATITIS	0.0250499	0.0446474	0.574757
rs11783093	Smoking status (ev	-0.025915962	0.00255102040816327	7e-22	finngen_R10_AB1_VIRAL_HEPATITIS	-0.0765518	0.0451202	0.0897677
rs117898875	Smoking status (ev	-0.031903576	0.00561224489795918	2e-08	finngen_R10_AB1_VIRAL_HEPATITIS	0.04561	0.0716894	0.524635
rs11844846	Smoking status (ev	-0.012888573	0.00229591836734694	2e-08	finngen_R10_AB1_VIRAL_HEPATITIS	-0.0827935	0.0297916	0.00545117
rs11872397	Smoking status (ev	-0.014873725	0.00255102040816327	2e-09	finngen_R10_AB1_VIRAL_HEPATITIS	0.0447853	0.0361834	0.215816
rs12211126	Smoking status (ev	-0.012434233	0.00204081632653061	1e-09	finngen_R10_AB1_VIRAL_HEPATITIS	0.000608079	0.0307982	0.984248
rs1221976	Smoking status (ev	0.014177998	0.00204081632653061	3e-11	finngen_R10_AB1_VIRAL_HEPATITIS	0.0854629	0.0334932	0.0107216
rs12456711	Smoking status (ev	-0.013501848	0.00244897959183674	4e-08	finngen_R10_AB1_VIRAL_HEPATITIS	-0.00301881	0.0344728	0.930218
rs1246265	Smoking status (ev	-0.015632885	0.00229591836734694	3e-11	finngen_R10_AB1_VIRAL_HEPATITIS	0.00810148	0.0330348	0.80627
rs12517438	Smoking status (ev	-0.011043831	0.00201530612244898	4e-08	finngen_R10_AB1_VIRAL_HEPATITIS	-0.0781037	0.0295229	0.00815624
rs1271272	Smoking status (ev	-0.011995592	0.00214285714285714	3e-08	finngen_R10_AB1_VIRAL_HEPATITIS	-0.0610737	0.0318931	0.0554983
rs13237637	Smoking status (ev	-0.016295116	0.00229591836734694	6e-14	finngen_R10_AB1_VIRAL_HEPATITIS	0.0403443	0.0297826	0.175536
rs13396935	Smoking status (ev	-0.019117456	0.00255102040816327	4e-13	finngen_R10_AB1_VIRAL_HEPATITIS	-0.00191875	0.0405286	0.96224
rs1373129	Smoking status (ev	0.012676746	0.00201530612244898	3e-10	finngen_R10_AB1_VIRAL_HEPATITIS	0.0192269	0.0296962	0.51734
rs1469908	Smoking status (ev	-0.014059351	0.00204081632653061	6e-12	finngen_R10_AB1_VIRAL_HEPATITIS	-0.0112132	0.0302756	0.711105
rs1492546	Smoking status (ev	0.014153526	0.00216836734693878	9e-11	finngen_R10_AB1_VIRAL_HEPATITIS	0.00668897	0.0297902	0.822341
rs16972552	Smoking status (ev	0.017533675	0.00331632653061225	2e-08	finngen_R10_AB1_VIRAL_HEPATITIS	-0.00847077	0.0562989	0.880401
rs17151637	Smoking status (ev	-0.014881184	0.00204081632653061	2e-11	finngen_R10_AB1_VIRAL_HEPATITIS	-0.00381826	0.0337008	0.909793
rs17348216	Smoking status (ev	-0.015646625	0.00280612244897959	4e-09	finngen_R10_AB1_VIRAL_HEPATITIS	-0.068532	0.0412545	0.0966741
rs176644	Smoking status (ev	-0.012165012	0.00204081632653061	3e-09	finngen_R10_AB1_VIRAL_HEPATITIS	0.0572334	0.0298805	0.0554396
rs1775370	Smoking status (ev	-0.013873694	0.00232142857142857	2e-09	finngen_R10_AB1_VIRAL_HEPATITIS	0.0551934	0.0425387	0.194463
rs1891196	Smoking status (ev	0.012412241	0.00204081632653061	9e-10	finngen_R10_AB1_VIRAL_HEPATITIS	0.00827857	0.0301957	0.783958
rs1909590	Smoking status (ev	-0.024852458	0.00306122448979592	4e-16	finngen_R10_AB1_VIRAL_HEPATITIS	-0.0115483	0.0360032	0.748393
rs2202237	Smoking status (ev	-0.013188412	0.00201530612244898	6e-11	finngen_R10_AB1_VIRAL_HEPATITIS	0.00968802	0.0300632	0.747259
rs2240294	Smoking status (ev	-0.012697397	0.00204081632653061	3e-10	finngen_R10_AB1_VIRAL_HEPATITIS	-0.00547321	0.0300582	0.855514
rs2310752	Smoking status (ev	-0.014862391	0.00204081632653061	2e-13	finngen_R10_AB1_VIRAL_HEPATITIS	-0.00319598	0.030696	0.917076
rs2340403	Smoking status (ev	-0.014000095	0.00209183673469388	2e-11	finngen_R10_AB1_VIRAL_HEPATITIS	-0.0411412	0.0309127	0.183228
rs2358443	Smoking status (ev	-0.011234026	0.00201530612244898	3e-08	finngen_R10_AB1_VIRAL_HEPATITIS	0.00281984	0.0301143	0.925397
rs240955	Smoking status (ev	-0.025524102	0.00280612244897959	4e-23	finngen_R10_AB1_VIRAL_HEPATITIS	-0.0447372	0.0328267	0.172936
rs2419903	Smoking status (ev	-0.014321689	0.00204081632653061	2e-12	finngen_R10_AB1_VIRAL_HEPATITIS	-0.0620812	0.0311898	0.0465436
rs2652434	Smoking status (ev	-0.011200736	0.00198979591836735	3e-08	finngen_R10_AB1_VIRAL_HEPATITIS	0.0174221	0.0297644	0.558324
rs28459916	Smoking status (ev	-0.01731356	0.00306122448979592	4e-08	finngen_R10_AB1_VIRAL_HEPATITIS	-0.00608411	0.0482307	0.899616
rs2866724	Smoking status (ev	-0.013502994	0.00224489795918367	3e-09	finngen_R10_AB1_VIRAL_HEPATITIS	-0.0526847	0.0318672	0.0982788
rs288175	Smoking status (ev	-0.012248558	0.00211734693877551	1e-08	finngen_R10_AB1_VIRAL_HEPATITIS	-0.0391762	0.0307798	0.203093
rs2939756	Smoking status (ev	-0.011942013	0.00201530612244898	3e-09	finngen_R10_AB1_VIRAL_HEPATITIS	0.00665739	0.0297842	0.82313
rs301805	Smoking status (ev	-0.011173298	0.00204081632653061	4e-08	finngen_R10_AB1_VIRAL_HEPATITIS	0.0166642	0.0313379	0.594892
rs3026629	Smoking status (ev	-0.011653287	0.00211734693877551	3e-08	finngen_R10_AB1_VIRAL_HEPATITIS	-0.0188109	0.0312488	0.547192
rs35761479	Smoking status (ev	-0.022095622	0.00331632653061225	3e-11	finngen_R10_AB1_VIRAL_HEPATITIS	-0.048086	0.0392205	0.220183
rs35891966	Smoking status (ev	-0.024323925	0.00433673469387755	7e-09	finngen_R10_AB1_VIRAL_HEPATITIS	-0.05051	0.0545422	0.354408
rs3800227	Smoking status (ev	-0.013393484	0.00229591836734694	5e-09	finngen_R10_AB1_VIRAL_HEPATITIS	-0.0249519	0.0318482	0.433354
rs3818987	Smoking status (ev	-0.013703931	0.00219387755102041	3e-10	finngen_R10_AB1_VIRAL_HEPATITIS	0.0195497	0.0307935	0.525517
rs3851186	Smoking status (ev	0.015413395	0.00280612244897959	7e-09	finngen_R10_AB1_VIRAL_HEPATITIS	-0.0628793	0.0353378	0.0751779
rs3980078	Smoking status (ev	0.015629334	0.00280612244897959	3e-08	finngen_R10_AB1_VIRAL_HEPATITIS	-0.0224771	0.0316595	0.477726
rs4312833	Smoking status (ev	-0.019415041	0.00357142857142857	4e-08	finngen_R10_AB1_VIRAL_HEPATITIS	0.0592835	0.0464241	0.201603
rs4543592	Smoking status (ev	0.012717051	0.00201530612244898	3e-10	finngen_R10_AB1_VIRAL_HEPATITIS	0.0470562	0.0304107	0.121777
rs4680392	Smoking status (ev	0.013638121	0.00232142857142857	4e-09	finngen_R10_AB1_VIRAL_HEPATITIS	0.0462324	0.0322267	0.151402
rs4737525	Smoking status (ev	-0.01328229	0.00201530612244898	4e-11	finngen_R10_AB1_VIRAL_HEPATITIS	-0.0449939	0.0299019	0.132397
rs4748779	Smoking status (ev	-0.013735452	0.00224489795918367	1e-09	finngen_R10_AB1_VIRAL_HEPATITIS	-0.0186159	0.0339156	0.583082
rs4856463	Smoking status (ev	-0.016243614	0.00255102040816327	7e-10	finngen_R10_AB1_VIRAL_HEPATITIS	-0.0482128	0.035272	0.171661
rs4869058	Smoking status (ev	-0.01564975	0.00204081632653061	8e-14	finngen_R10_AB1_VIRAL_HEPATITIS	-0.0638803	0.0314198	0.0420397
rs4949465	Smoking status (ev	-0.01833924	0.00280612244897959	4e-10	finngen_R10_AB1_VIRAL_HEPATITIS	0.00683361	0.039399	0.862301
rs4984916	Smoking status (ev	-0.017400982	0.00229591836734694	7e-13	finngen_R10_AB1_VIRAL_HEPATITIS	-0.0776425	0.0313232	0.0131841
rs6265	Smoking status (ev	-0.02215149	0.00255102040816327	4e-18	finngen_R10_AB1_VIRAL_HEPATITIS	-0.0489878	0.0413796	0.236467
rs6438208	Smoking status (ev	-0.012643845	0.00232142857142857	4e-08	finngen_R10_AB1_VIRAL_HEPATITIS	0.00484238	0.0334707	0.884967
rs6474609	Smoking status (ev	-0.01275978	0.00221938775510204	7e-09	finngen_R10_AB1_VIRAL_HEPATITIS	0.0135279	0.0297975	0.649832
rs6598539	Smoking status (ev	0.012742009	0.00216836734693878	4e-09	finngen_R10_AB1_VIRAL_HEPATITIS	0.0417253	0.0297727	0.161076
rs6720647	Smoking status (ev	-0.013628829	0.0023469387755102	7e-09	finngen_R10_AB1_VIRAL_HEPATITIS	-0.0630953	0.0372616	0.0903982
rs6965740	Smoking status (ev	-0.013907693	0.00201530612244898	5e-12	finngen_R10_AB1_VIRAL_HEPATITIS	0.0143392	0.0308397	0.641962
rs6971782	Smoking status (ev	-0.011877323	0.00214285714285714	3e-08	finngen_R10_AB1_VIRAL_HEPATITIS	0.0335672	0.0355958	0.345676
rs7092200	Smoking status (ev	-0.019713078	0.00204081632653061	4e-22	finngen_R10_AB1_VIRAL_HEPATITIS	-0.0330088	0.0305143	0.279364
rs7124348	Smoking status (ev	-0.013640967	0.00237244897959184	9e-09	finngen_R10_AB1_VIRAL_HEPATITIS	0.00855576	0.0341081	0.801936
rs7160389	Smoking status (ev	-0.011882559	0.00201530612244898	4e-09	finngen_R10_AB1_VIRAL_HEPATITIS	-0.0435534	0.0298662	0.144763
rs72678864	Smoking status (ev	-0.018037062	0.00306122448979592	3e-10	finngen_R10_AB1_VIRAL_HEPATITIS	-0.0526921	0.0434007	0.224715
rs7295765	Smoking status (ev	-0.014850353	0.00267857142857143	4e-08	finngen_R10_AB1_VIRAL_HEPATITIS	0.0097752	0.039278	0.80346
rs7333559	Smoking status (ev	-0.015744165	0.00255102040816327	3e-09	finngen_R10_AB1_VIRAL_HEPATITIS	-0.118924	0.038956	0.00226741
rs76608582	Smoking status (ev	-0.033407107	0.00510204081632653	6e-11	finngen_R10_AB1_VIRAL_HEPATITIS	-0.0295185	0.064012	0.644699
rs7668995	Smoking status (ev	-0.016343247	0.00229591836734694	1e-13	finngen_R10_AB1_VIRAL_HEPATITIS	-0.00733311	0.0324591	0.821265
rs772921	Smoking status (ev	-0.012490748	0.00211734693877551	4e-09	finngen_R10_AB1_VIRAL_HEPATITIS	0.0077438	0.0318304	0.807786
rs7754741	Smoking status (ev	-0.015288658	0.00229591836734694	1e-11	finngen_R10_AB1_VIRAL_HEPATITIS	0.044474	0.031601	0.159321
rs77878475	Smoking status (ev	-0.025840854	0.0038265306122449	3e-11	finngen_R10_AB1_VIRAL_HEPATITIS	-0.0011375	0.0510085	0.982208
rs78175438	Smoking status (ev	-0.018702744	0.00331632653061225	8e-09	finngen_R10_AB1_VIRAL_HEPATITIS	0.0523992	0.0455398	0.249887
rs7870475	Smoking status (ev	-0.012688204	0.00201530612244898	3e-10	finngen_R10_AB1_VIRAL_HEPATITIS	-0.084129	0.0297867	0.00473729
rs7901883	Smoking status (ev	-0.013077027	0.00239795918367347	4e-08	finngen_R10_AB1_VIRAL_HEPATITIS	0.0822637	0.0350178	0.018814
rs7921378	Smoking status (ev	-0.017524462	0.00178571428571429	3e-18	finngen_R10_AB1_VIRAL_HEPATITIS	0.0254429	0.0297575	0.392546
rs7938812	Smoking status (ev	0.029930087	0.00204081632653061	7e-48	finngen_R10_AB1_VIRAL_HEPATITIS	-0.0141131	0.0309322	0.648204
rs9388686	Smoking status (ev	0.01442028	0.00262755102040816	4e-08	finngen_R10_AB1_VIRAL_HEPATITIS	-0.00299967	0.0374746	0.936201
rs9571576	Smoking status (ev	-0.011463561	0.00201530612244898	1e-08	finngen_R10_AB1_VIRAL_HEPATITIS	-0.0187781	0.0298882	0.529822
rs961414	Smoking status (ev	-0.022034988	0.00204081632653061	7e-28	finngen_R10_AB1_VIRAL_HEPATITIS	-0.0201893	0.0299729	0.500575
rs962472	Smoking status (ev	-0.011598815	0.00198979591836735	8e-09	finngen_R10_AB1_VIRAL_HEPATITIS	-0.0345876	0.0297966	0.245727

Instrument Variables for MR analysis of Smoking cessation and viral hepatitis

SNP	exposure	beta.exposure	se.exposure	pval.exposure	outcome	beta.outcome	se.outcome	pval.outcome
rs1006365	Smoking cessation	-0.00876314	0.00155612244897959	1e-08	finngen_R10_AB1_VIRAL_HEPATITIS	0.00472819	0.0298245	0.874036
rs10922907	Smoking cessation	-0.00936816	0.00155612244897959	2e-09	finngen_R10_AB1_VIRAL_HEPATITIS	-0.110018	0.030891	0.00036874
rs11756673	Smoking cessation	-0.0117212	0.00158163265306122	2e-13	finngen_R10_AB1_VIRAL_HEPATITIS	-0.00167669	0.0320074	0.958222
rs12203592	Smoking cessation	-0.0116721	0.00204081632653061	9e-09	finngen_R10_AB1_VIRAL_HEPATITIS	0.0377688	0.0894965	0.673015
rs12378015	Smoking cessation	-0.013593	0.00178571428571429	5e-16	finngen_R10_AB1_VIRAL_HEPATITIS	-0.136187	0.0402415	0.000713773
rs12969480	Smoking cessation	-0.00975168	0.00176020408163265	4e-08	finngen_R10_AB1_VIRAL_HEPATITIS	-0.042034	0.0358888	0.241507
rs1392816	Smoking cessation	-0.0118095	0.00158163265306122	2e-13	finngen_R10_AB1_VIRAL_HEPATITIS	0.0270062	0.031391	0.389614
rs1475065	Smoking cessation	-0.0116659	0.00165816326530612	1e-12	finngen_R10_AB1_VIRAL_HEPATITIS	-0.0405543	0.0319717	0.204639
rs1529675	Smoking cessation	-0.0104344	0.00158163265306122	5e-11	finngen_R10_AB1_VIRAL_HEPATITIS	-0.000260615	0.0301794	0.99311
rs1565735	Smoking cessation	-0.0167706	0.00204081632653061	3e-18	finngen_R10_AB1_VIRAL_HEPATITIS	-0.0631011	0.0422273	0.135092
rs2098651	Smoking cessation	-0.0106521	0.00155612244897959	2e-11	finngen_R10_AB1_VIRAL_HEPATITIS	-0.0639857	0.0310965	0.0396242
rs2421667	Smoking cessation	-0.0100053	0.00158163265306122	4e-10	finngen_R10_AB1_VIRAL_HEPATITIS	0.0128121	0.0308904	0.678317
rs2424880	Smoking cessation	-0.00881264	0.00155612244897959	1e-08	finngen_R10_AB1_VIRAL_HEPATITIS	0.000594585	0.0306893	0.984543
rs264952	Smoking cessation	-0.0115881	0.00153061224489796	5e-14	finngen_R10_AB1_VIRAL_HEPATITIS	0.029195	0.0299696	0.329982
rs2734838	Smoking cessation	-0.0104198	0.00155612244897959	3e-11	finngen_R10_AB1_VIRAL_HEPATITIS	-0.0248864	0.0298335	0.404182
rs2754511	Smoking cessation	-0.00925063	0.00165816326530612	2e-08	finngen_R10_AB1_VIRAL_HEPATITIS	-0.0155688	0.0309758	0.615238
rs3025388	Smoking cessation	-0.0166063	0.00204081632653061	3e-16	finngen_R10_AB1_VIRAL_HEPATITIS	-0.046619	0.0415559	0.261932
rs329120	Smoking cessation	-0.0114057	0.00153061224489796	2e-13	finngen_R10_AB1_VIRAL_HEPATITIS	0.0286897	0.0301261	0.340933
rs3820277	Smoking cessation	-0.00852278	0.00155612244897959	5e-08	finngen_R10_AB1_VIRAL_HEPATITIS	0.00239064	0.0298419	0.936149
rs4705014	Smoking cessation	-0.0095009	0.00158163265306122	3e-09	finngen_R10_AB1_VIRAL_HEPATITIS	-0.0595897	0.030575	0.0512991
rs4783722	Smoking cessation	-0.00942456	0.00155612244897959	2e-09	finngen_R10_AB1_VIRAL_HEPATITIS	0.00540319	0.0300159	0.857144
rs56099375	Smoking cessation	-0.0103936	0.00183673469387755	2e-08	finngen_R10_AB1_VIRAL_HEPATITIS	-0.0328857	0.0367486	0.370849
rs56113850	Smoking cessation	-0.00974331	0.00155612244897959	6e-10	finngen_R10_AB1_VIRAL_HEPATITIS	-0.0307794	0.0305233	0.313267
rs591143	Smoking cessation	-0.0136814	0.00153061224489796	3e-18	finngen_R10_AB1_VIRAL_HEPATITIS	0.0193821	0.0313572	0.536507
rs59617050	Smoking cessation	-0.0113382	0.00155612244897959	6e-13	finngen_R10_AB1_VIRAL_HEPATITIS	-0.035432	0.0299343	0.236548
rs6011779	Smoking cessation	-0.0260499	0.00204081632653061	1e-39	finngen_R10_AB1_VIRAL_HEPATITIS	-0.0232692	0.0345485	0.500615
rs60749569	Smoking cessation	-0.0208199	0.00306122448979592	8e-13	finngen_R10_AB1_VIRAL_HEPATITIS	0.00264967	0.0743502	0.971571
rs62369913	Smoking cessation	-0.0127382	0.00204081632653061	5e-10	finngen_R10_AB1_VIRAL_HEPATITIS	-0.0361956	0.0440022	0.410743
rs6425280	Smoking cessation	-0.00976468	0.00158163265306122	7e-10	finngen_R10_AB1_VIRAL_HEPATITIS	0.00534565	0.0298495	0.857869
rs6452784	Smoking cessation	-0.0092739	0.00153061224489796	2e-09	finngen_R10_AB1_VIRAL_HEPATITIS	-0.0223893	0.029767	0.45196
rs6737187	Smoking cessation	-0.0108357	0.00168367346938776	1e-10	finngen_R10_AB1_VIRAL_HEPATITIS	-0.0469955	0.0325076	0.148268
rs6756212	Smoking cessation	-0.0116286	0.00155612244897959	6e-14	finngen_R10_AB1_VIRAL_HEPATITIS	-0.0206105	0.0299672	0.4916
rs6968125	Smoking cessation	-0.00912677	0.00155612244897959	4e-09	finngen_R10_AB1_VIRAL_HEPATITIS	-0.0173163	0.0299114	0.562643
rs72678864	Smoking cessation	-0.012035	0.00211734693877551	1e-08	finngen_R10_AB1_VIRAL_HEPATITIS	-0.0526921	0.0434007	0.224715
rs73178638	Smoking cessation	-0.0152026	0.00204081632653061	1e-14	finngen_R10_AB1_VIRAL_HEPATITIS	-0.0129489	0.0350648	0.711915
rs75503803	Smoking cessation	-0.0140337	0.00232142857142857	1e-09	finngen_R10_AB1_VIRAL_HEPATITIS	-0.0351285	0.0456714	0.4418
rs7689452	Smoking cessation	-0.0103017	0.00163265306122449	4e-10	finngen_R10_AB1_VIRAL_HEPATITIS	-0.0255968	0.030847	0.406653
rs7778443	Smoking cessation	-0.0131575	0.00153061224489796	7e-17	finngen_R10_AB1_VIRAL_HEPATITIS	-0.00701046	0.0329437	0.831482
rs79932649	Smoking cessation	-0.0183902	0.00331632653061225	4e-09	finngen_R10_AB1_VIRAL_HEPATITIS	0.00523977	0.053538	0.922035
rs9409844	Smoking cessation	-0.0273437	0.0038265306122449	3e-13	finngen_R10_AB1_VIRAL_HEPATITIS	-0.0966734	0.0706967	0.171488
rs9651873	Smoking cessation	-0.0109714	0.00155612244897959	2e-12	finngen_R10_AB1_VIRAL_HEPATITIS	0.0511551	0.0300732	0.0889385

© 2025 Lin B. et al.
